# Validity of Energy Intake Reports in Relation to Dietary Patterns

**Published:** 2014-03

**Authors:** Mahboubeh Shaneshin, Mahsa Jessri, Bahram Rashidkhani

**Affiliations:** ^1^Community Nutrition Department, Faculty of Nutrition Sciences and Food Technology, National Nutrition and Food Technology Research Institute, Shahid Beheshti University of Medical Sciences and Health Services, Tehran, Iran;; ^2^Department of Nutritional Sciences, Faculty of Medicine, University of Toronto, Toronto, ON, Canada, M5S 3E2

**Keywords:** Dietary patterns, Energy underreporting, Validity, Women, Iran

## Abstract

The role of under- and overreporting of energy intake in determining the dietary patterns is yet unclear, especially in the Middle Eastern countries. This study identifies the prevalence of misreporting among Tehranian women aged 18-45 years and to compare the dietary intake patterns of plausible and all energy reporters. Dietary intakes and anthropometric data were collected. FitMate™ metabolic analyzer and Goldberg equation were used in determining the under/overreporting of energy intake. Underreporters were more likely to be overweight and older compared to plausible reporters. Three dietary patterns emerged for all reporters, and two were identified for plausible reporters. Using only plausible reporters to determine dietary patterns was not similar to using all reporters. The proportion of underreporters was 59.3% in the mixture cluster, 30.4% in the unhealthy cluster, and 35.3% in the healthy cluster (p<0.05). Underreporting of energy intake is not uniformly distributed among dietary pattern clusters and tends to be less severe among subjects in the unhealthy cluster. Our data suggested that misreporting of energy intake might affect the dietary pattern analysis.

## INTRODUCTION

The association between diet and health outcomes is considered one of the most challenging areas of nutritional epidemiology (1,2). Food consumption surveys mainly concern this relationship (3). It has been widely proven that the intrinsic inaccuracy of dietary intake assessment methods, i.e. 24-hour dietary recalls, food records and food frequency questionnaire (FFQ) is the main cause of uncertainty regarding the role of diet in aetiology of chronic diseases (4,5). Dietary assessment methods are based on self-reported dietary intake, which is liable to potential biases and often underestimates the energy intake (3,6-9).

For about three decades, the accuracy of dietary assessment methods has been assessed by the doubly-labelled water (DLW) as the ‘gold standard’ marker of the validity of self-reports on energy intake (EI) (10). This method is not feasible to be used in large epidemiological studies due to its cost and complexity (3,11). A simple method developed by Goldberg *et al*. is alternatively used for identifying subjects who remarkably under- or overreport their energy intakes, by dividing their energy intake to basal metabolic rate (EI:BMR) (1,5,12-14). Current data in the developing countries show a vast range of underreporting and, in Iran, this phenomenon has been proven to be most prevalent in urban adult population and is highly associated with sex, age, and body mass index (BMI) (1).

In assessment of health status, considering the overall dietary pattern rather than single nutrients or food items is preferred (15-21) since the association of diet and disease will end up being oversimplified when a single nutrient is analyzed separately (22). Even more problematic is the fact that underreporting is not random, and underreporters tend to estimate low intakes of foods perceived as unhealthy (3,23), which may lead to a heavily-biased interpretation of diet-disease interaction (2). However, few studies have concerned the role of inaccuracy of energy intake based on self-reporting of the food-group intakes in the emergence of disease outcomes (13,24,25). Bailey *et al*. determined two dietary patterns in a sample of 179 elderly individuals for all and plausible reporters: healthy and unhealthy. They showed that using only plausible reporters to determine dietary patterns yielded very similar results compared to using all reporters (11). Scagliusi *et al*. studied whether underreporting rates vary among dietary pattern clusters in Brazilian women, and they identified three dietary pattern clusters: sweet foods, starchy foods, and healthy foods (22). Underreporting of EI was not uniformly distributed among dietary pattern clusters and tended to be more severe among subjects from the healthy cluster (22). A better understanding of the food habits of underreporters will help reduce the bias caused by reporting errors in nutritional surveys (3). The objectives of the present study were, therefore, to identify misreporting among a sample of Iranian women and to investigate the effect of the reporting errors on dietary patterns.

## MATERIALS AND METHODS

### Population

This cross-sectional study was conducted on 187 Iranian women aged 18-45 years, residing in Tehran, Iran (26). Participants were chosen based on a stratified random-sampling method from all regions of the Tehran municipality. The sampling strategy of the study followed a stratified random-sampling procedure with proportional allocation within strata. The stratification of the sample according to the 22 municipal districts of Tehran city is incorporated in the sampling process. An appropriate number of health centres were assigned to each of the 22 districts. The second step was to take a simple random sample within each centre. A total of 232 women were invited to participate in this study, of whom, 210 agreed to participate (response rate: 90%). Subjects with any clinical disorders (e.g. liver, kidney, endocrine) or those who were taking medications which affected the resting metabolic rate (RMR) (e.g. antidepressants, beta-blockers, hormones, etc.) were excluded from the study (n=23). None of the subjects had dieting history and overt weight changes at least for the past two months. The objectives and the procedures were fully described individually for all subjects before taking written informed consents. The protocol and procedures of this study were approved by the Ethics Committee of the National Nutrition and Food Technology Research Institute, Shahid Beheshti University of Medical Sciences and Health Services, Iran.

### Anthropometric and dietary assessment

Trained dietitians performed all anthropometric and dietary intake assessments. Body-weight was measured to the nearest 0.1 kg while subjects were minimally clothed and standing on digital scales (Soehnle, Germany) without shoes. Height was measured to the nearest 0.5 cm, using a non-stretch tape-meter fixed to a wall, with subjects standing without shoes while shoulders were in a normal position. Body mass index (BMI) was calculated by dividing the weight in kg by square of height in metre (kg/m^2^).

For collection of dietary data, a 168-item semi-quantitative food frequency questionnaire (FFQ) validated for Iranian population was used **(27)**. This FFQ is a Willet-format questionnaire modified based on Iranian food items and contains questions on average food consumption and intake frequency during the previous year. The food items included in the FFQ have been chosen according to the most frequently-consumed items in the national food consumption surveys in Iran. Because different recipes are used for food preparation, the FFQ is based on food items rather than dishes (e.g. beans, different meats, oils, and rice). Subjects indicated their food consumption frequencies on a daily, weekly, monthly, and yearly basis according to portion-sizes provided in the FFQ. For each food item in the FFQ, a portion-size was specified using USDA serving sizes (e.g. bread-1 slice; apple-1 medium; dairy-1 cup) whenever possible, and if this was not possible, household measures (e.g. beans-1 tablespoon; chicken meat-1 leg, breast, or wing; rice-1 large, medium, or small plate) were chosen alternatively. Because the only available Iranian food composition table (FCT) analyzes a very limited number of raw food items and nutrients (28), we used the USDA's FCT (29) alternatively. The Iranian FCT was used only for estimating traditional Iranian food items, like *kashk*, which are not included in the USDA's FCT. The reported dietary intakes were then converted to daily grammes of food intake by using household measures (30). Food items included in the FFQ were classified into 39 predefined food-groups (31) based on the similarity of their nutrient profiles or culinary usage of the foods.

### Measurement of resting metabolic rate

Since accurate measurement of RMR typically needs incorporating competent technicians and complicated costly methodologies, its measurement is impractical in most clinical and community settings (32). Prediction equations use usual variables, such as age, sex, height, and body mass to predict RMR, although these can only predict 50-75% of variability in RMR (33,34). New portable devices for measuring RMR are less costly and easier to use compared to traditionally-used metabolic carts. Cosmed has recently developed a small (20×24 cm) metabolic analyzer (FitMate™) to assess oxygen and energy consumption during rest and exercise. The Cosmed Fitmate PRO is a dynamic and cost-effective desktop testing device that provides oxygen consumption by dynamic mixing chamber and measure VO_2_, minute ventilation, heart rate, and related parameters with a 15-second sampling rate. Fitmate PRO offers most of the features of conventional metabolic carts but at incomparable affordable costs. Quality control is done during testing (leaks on the mask, HR missing, non-physi­ological gas measurements, etc.). The FitMate™ metabolic system used in this study had a good relative validity and was reproducible for measuring RMR and appeared to be an acceptable tool for measuring RMR in adults. In a previous study, RMR was measured simultaneously with the Douglas bag and FitMate™ systems. No significant differences were found between Douglas bag and FitMate™ systems for oxygen consumption (p=0.07, r=0.97) and RMR values (p=0.58, r=0.97) (32). In the present study, FitMate™ calorimeter (Cosmed, Rome, Italy) was used by a trained nutritionist for measuring RMR according to the manufacturer's protocol. Tests were performed between 8 and 9 am and after 12 hours of fasting. Subjects were instructed to have light evening meal between 19:30 and 20:00 in the previous day. During the 24-hour period prior to the study, subjects abstained from exercise. Participants also refrained from smoking, alcohol, caffeine, and drugs for 12 hours before the study. Prior to measuring RMR, subjects stayed supine for 25±30 min in a quiet room with a temperature between 22 ºC and 24 ºC.

During the procedure, the subject was in a supine position with a mask fully covering her nose and mouth to measure oxygen consumption (VO_2_) for 15 minutes. Ventilation rate was measured using a flow-meter, and the fraction of oxygen in expired gases was assessed using a galvanic fuel cell oxygen sensor. RMR was then calculated from oxygen consumption, using a fixed respiratory quotient (RQ) of 0.85 and estimated grammes of urinary nitrogen from a modified Weir equation as follows (35):

Weir equation: REE=[O_2_ consumed (litre) x 3.9 produced CO_2_ (litre) x 1.1] x 1,440 min/d, where REE= resting energy expenditure, d=day, min=minute.

### Definition of underreporters of energy intake

We calculated the EI:RMR ratio to evaluate the validity of energy intake. To compare the relative degree of under- and overreporting, we temporarily used the values defined by Goldberg *et al*. (12). Goldberg *et al*. have calculated the minimum energy requirement by whole body calorimetry and coefficients for physical activity levels suggested by FAO/WHO/UNU (36) for estimating the energy expenditure. The results of their survey show that the energy intake (EI) to RMR ratio of <1.35 is not consistent with usual dietary intake. So, an EI:RMR value of 1.35 was suggested as a cutoff that is compatible with a normal, not bedfast lifestyle (13) and, thus, less than this cutoff was considered underreporting. Black *et. al*. suggested overreporting to be the EI:RMR ratio of ≥2.4 (37) as the maximum for a sustainable lifestyle (13). These cutoff values do not take into account the true energy expenditure of each individual. Therefore, in the present study, a range of 1.35-2.39 is considered normal reporting of energy intake.

### Assessment of other variables

Age, history of any disease, taking any medications, diet counselling from a physician or dietitian, and weight change during the past two months were inquired by trained interviewers. Information on socioeconomic and lifestyle factors was collected from the lifestyle multiple-choice questionnaire.

### Statistical analysis

All statistical analyses were performed using the Statistical Package for Social Sciences (SPSS Inc. Chicago, IL, version 16), and the significance level was defined at p=0.05. Factor and cluster analyses are the main data-driven methods to study dietary patterns. Cluster analysis identifies mutually-exclusive groups of individuals, unlike factor analysis, which reduces dietary data into patterns based on correlation between foods. Cluster solutions may vary depending on strategies used. We performed the cluster analysis on daily intakes of food-groups in grammes per 1,000 kcal. This correction for energy intake prevents big eaters from exerting undue influence on the resulting patterns (38).

We used SPSS QUICK CLUSTER procedure (K-means Method) that uses the Euclidean distances between observations to empirically estimate clusters. The itineration was employed (its maximum number was 10). Subjects are assigned exclusive cluster membership based on the Euclidean distance of data points from the cluster centroid in an iterative process. Thus, a cluster represents a dietary pattern of a group of individuals based on similarity of consumption of food-groups. We chose a solution with two and three clusters because it provided more separated clusters (according to the ANOVA that compared the variables of the food-groups between the clusters, for each solution) and greater ease in interpreting results. A cluster solution with 2 (among plausible reporters) or 3 clusters (among all reporters) included a clear ‘healthy cluster’ whereas the analysis with more or less potential clusters included clusters that could be so difficult to interpret in some aspects. Therefore, the solution with 2 or 3 clusters was selected.

One-way analysis of variance (ANOVA) was used for determining the differences between multiple means in continuous variables while chi-square test was used for comparing the proportion of underreporters between dietary pattern clusters.

## RESULTS

Of the one hundred and eighty-seven participants, 35.3% were underreporters (EI:RMR ratio of <1.35), and 7.4% were overreporters (EI:RMR ratio of ≥2.4). The mean age of participants was 35 years, and the average EI:RMR ratio was 1.6:0.6. Compared to plausible reporters, underreporters had higher BMIs and were significantly older (p<0.05). Also, severe underreporters were mostly aged ≥30 years and obese (BMI >30). Overreporting of energy intake was significantly associated with having lower RMR (p<0.01); and overreporters as a group were significantly younger and had a lower BMI compared to plausible reporters (Table 1). There was no difference between underreporters, overreporters, and plausible reporters in regard to educational level, smoking, desire for weight reduction, and socioeconomic status.

Table 2 presents the mean intake from each food-group by cluster of diet pattern in all reporters and plausible reporters. Regarding the energy intake, underreporters reported, on average, 632 kcal less than plausible reporters (p<0.01) (data not shown). When the dietary pattern of all reporters was compared with those of plausible reporters, significant differences were observed (Table 2). Three dietary patterns were determined for all reporters according to their most recurrent food-group intake. The first cluster consisted of 27 subjects and was named ‘mixture food’ which included high consumption of tea and low-fat dairy products. The second cluster was named ‘unhealthy cluster’ (125 participants) and was characterized by low consumption of grain, potato, green vegetables, other vegetables, tomato, fruits, poultry, nuts, dry fruits, low-fat dairy products, and yogurt drink. Healthy food cluster was composed of all foods of the abovementioned food-groups as well as fish and sauce. Also, two dietary patterns were reported for plausible reporters, namely healthy and unhealthy food clusters. The first cluster included high consumption of potato, yellow vegetables, other vegetables, and tea while the second cluster was composed of low consumption of potato, yellow vegetables, other vegetables, and tea.

Table 2 shows that the mean intakes from high consumption of grain, potato, green vegetables, other vegetables, tomato, fruits, poultry, fish, nuts, dry fruits, yogurt drink, low-fat dairy products, and sauce are significantly higher in ‘healthy food cluster’ compared to ‘unhealthy food cluster’ (p<0.01). On the other hand, consumption of tea and low-fat dairy products was significantly higher in the mixture food cluster compared to the healthy and unhealthy food cluster (p<0.0001). Plausible reporters in the healthy food cluster tend to consume more of other vegetables, potato, yellow vegetables, and tea compared to participants of unhealthy food cluster (p<0.05). The underlined food subgroups, namely potato and other vegetables, were consistent across the healthy/unhealthy clusters in both plausible and all reporters (p<0.01).

Table 3 shows the characteristics of participants in each dietary pattern cluster. We found three clusters, with most people being in the unhealthy cluster (67%), followed by healthy cluster (19%) and mixture cluster (14%). Among participants, underreporting of energy was not uniformly distributed among dietary pattern clusters. The proportion of underreporters was 59.3% in the mixture cluster, 30.4% in the unhealthy cluster, and 35.3% in the healthy cluster (p<0.05); As presented, height, EI, and EI:RMR ratio were significantly associated with food-group clusters among all energy reporters (p≤0.01). Height had the highest value among unhealthy food cluster, EI and the EI:RMR ratio were the highest among unhealthy food cluster, too. The educational level of healthy cluster was higher compared to unhealthy cluster (p=0.02). There was no difference between mixture food, healthy food, and unhealthy food clusters in socioeconomic status.

## DISCUSSION

This study showed the effect of reporting errors on patterns of diet intake among a representative sample of Tehranian women. More than one-third of the study participants were classified as underreporters while less than 10% were considered overreporters. Underreporters were shown to be older and with higher BMI compared to plausible reporters. Among Tehranian women, underreporting of energy intake is not uniformly distributed among dietary pattern clusters and tends to be less severe among subjects from the unhealthy cluster. The underreporting of energy did alter the dietary pattern found within this sample.

**Table 1.** Descriptive characteristics of the reporters of energy intake among Tehranian women (N=187)

Parameter	Underreporter†† (n=66)	Plausible reporter (n=107)	Overreporter (n=14)	p value
Age (years)	36±8‡‡,*	35±10	29±9	0.01
Body-weight (kg)	73±17***	66±15	61±8	0.001
Height (cm)	155±20	155±21	157±7	0.87
BMI† (kg/m^2^)	29±6*	27±6	25±5	0.007
EI‡ (kcal/day)	1,774±463***	2,406±472	3,711±1049***	0.0001
(kcal/day) § RMR	1,652±293***	1,393±238	1,171±179**	0.0001
BMI (kg/m^2^) (%)				
<25	15 (23)	40 (37)	8 (58)	
25-30	19 (29)	35 (33)	3 (21)	0.03
>30	32 (48)	32 (30)	3 (21)	
Age (years)				
<30	14 (21)	32 (30)	9 (64)	
30-40	24 (36)	33 (31)	3 (22)	0.04
>40	28 (42)	42 (39)	2 (14)	

†Body mass index;

‡Energy intake;

1 Resting metabolic rate;

2 Underreporter: EI:RMR ratio≥1.34, Plausible reporter: 1.34<EI:RMR<2.4, Overreporter: EI:RMR ≤2.4;

3 Values are expressed as mean±standard deviation or n (%);

Significantly different from plausible reporters:

*p<0.05

**p<0.01

***p<0.001

One-way ANOVA or Kruskal-Wallis and chi-square test

The prevalence of underreporting in this study is in line with other studies conducted in developing countries reporting a value ranging from 21% to 45% (13,39-41), and overreporting ranging from 5 to 7% (13). However, few studies have concerned this issue in developing countries. In Jamaica, an underreporting value of 38.6% in women and 22.5% in men have been reported (2) while two previous studies in South Africa (42) and Egypt (13) reported a rate of 43% and 10% respectively. These discrepancies may be attributed to the methodological differences (such as approaches used for identifying underreporting), dietary assessment techniques used, and cultural differences (2). To our knowledge, no previous study in Iran assessed the prevalence of abnormal eating attitude among Iranian women. In fact, we do not know the prevalence of Iranian women who are self-conscious about their diet. A cutoff point of energy intake <1.35×RMR was used for identifying underreporters as in other studies. Based on this cutoff, 35.3% of our subjects were underreporters. This was very similar to the levels reported in developing countries, such as Jamaica (2) and South Africa (42).

We found underreporters to be older and with higher BMI compared to plausible reporters. Studies conducted in both developing (1,2,43) and industrialized countries (44) showed similar findings.

Although understanding the demographic and general characteristics of underreporters is important, assessing the effect of implausible reporting on food-group intake and dietary pattern derivation is equally critical (11). Implausible reporters have been shown to selectively underreport foods perceived as unhealthful (such as sweets, fats, and snacks) (3,9,10,40,45). Also, food items considered ‘bad for health’ (e.g. butter, French fries) are preferably less reported by underreporters than foods considered ‘good for health’ (e.g. vegetables, fat-reduced products) (3). Holmbäck *et al.* reported that a large proportion of women was classified as underreporters in the coffee pattern group (46). In the present study, underreporting was more prevalent among the ‘mixture food’ which included high consumption of tea. It is possible that some women report having only coffee or tea and neglect to report an accompanying snack or cake, leading to misclassification of this meal type. Furthermore, Holmbäck *et al.* reported that, among women, the cake pattern had the lowest proportion of underreporters, suggesting that individuals reporting a high frequency of cake and biscuit meals do not care reporting on eating ‘unhealthy’ foods (46). This is in agreement with our finding that the lowest proportion of underreporters was among unhealthy pattern.

**Table 2.** Mean food-group intake (g/day) among Tehranian women for plausible and all reporters of energy intake†,‡

Food-group	All reporters (n=187)			p value§	Plausible reporters (n=107)			p value§
	Cluster 1: Mixture food (n=27)	Cluster 2: Unhealthy food (n=125)	Cluster 3: Healthy food (n=35)		Cluster 1: Healthy food (n=36)	Cluster 2: Unhealthy food (n=71)		
Grain	13.3 (9.6-17)	12.4 (10.3-14.4)	22.0 (15.4-28.7)	0.001	115.5 (93.6-136.6)	90.5 (81.1-99.9)	0.71
Potato	13.8 (8.3-19.3)	8.9 (7.3-10.6)	15.2 (9.3-21.0)	0.01	13.7 (9.5-17.8)	8.2 (5.5-11.0)	0.03
Green vegetable	16.6 (7.4-25.8)	8.1 (6.4-9.8)	18.1 (13.0-23.3)	0.0001	9.8 (7.1-12.4)	9.1 (6.7-11.4)	0.71
Other vegetables	134.2 (103.0-165.4)	83.1 (76.3-90.0)	148.9 (126.2-171.6)	0.0001	115.1 (93.6-136.6)	90.5 (81.1-99.9)	0.02
Tomato	70.6 (34.4-106.7)	59.8 (51.7-67.9)	100.2 (77.9-122.5)	0.002	60.9 (43.3-78.5)	71.1 (58.3-83.9)	0.35
Fruits	248.2 (174.5-321.9)	159.2 (146.4-172.0)	389.1 (345.3-432.8)	0.0001	214.6 (161.3-267.9)	215.5 (192.6-238.5)	0.97
Dry fruits	8.2 (3.9-12.4)	5.4 (4.4-6.4)	10.5 (7.1-13.9)	0.001	6.8 (4.1-9.5)	7.4 (5.6-9.2)	0.70
Fish	2.7 (1.4-4.1)	3.6 (3.1-4.2)	5.2 (3.3-7.0)	0.03	4.2 (2.8-5.6)	4.4 (3.5-5.3)	0.86
Poultry	7.7 (5.6-9.7)	6.0 (5.1-6.9)	9.3 (7.2-11.5)	0.004	6.9 (5.2-8.7)	6.0 (4.9-7.1)	0.36
Nuts	4.3 (2.2-6.3)	2.7 (2.2-3.1)	4.4 (2.1-6.6)	0.03	3.5 (2.2-4.9)	3.0 (2.3-3.7)	0.45
Low-fat dairy	184.2 (115.9-252.4)	78.9 (66.8-91.1)	159.5 (114.1-204.9)	0.0001	100.3 (75.3-125.4)	102.0 (80.4-123.7)	0.92
Yogurt drink	48.4 (24.8-72.0)	44.3 (33.9-55.7)	122.0 (78.8-165.2)	0.0001	41.5 (22.1-61.0)	67.8 (49.3-86.3)	0.08
Sauce	11.0 (5.7-16.4)	12.9 (10.0-15.9)	23.6 (13.1-34.0)	0.01	10.9 (7.1-14.6)	16.5 (12.5-20.5)	0.08
Tea	746.0 (674.7-817.2)	242.9 (217.1-266.8)	219.7 (175.6-263.8)	0.0001	489.0 (439.9-538.1)	186.0 (163.0-20.9)	0.0001
Yellow vegetable	10.5 (7.0-13.9)	9.2 (6.9-11.6)	12.5 (8.2-16.9)	0.40	12.4 (7.1-17.6)	6.8 (4.4-9.0)	0.03

†Values are expressed as mean (CI);

‡Underlined food items represent consistent findings with all reporters and plausible reporters;

§p values were obtained by means of ANOVA test that compared daily grammes of intake of food groups between the clusters; Figures in parentheses are 95% ranges

**Table 3.** Characteristics of the subjects according to each dietary pattern cluster

Variable	All reporters (n=187)		
	Cluster 1: Mixture food (n=27)		Cluster 2: Unhealthy food (n=125)		Cluster 3: Healthy food (n=35)		p values
	Mean (%)	95% CI	Mean (%)	95% CI	Mean (%)	95% CI
Age (years)	34.3	31.0-37.6	34.5	33.1-36.0	36.8	34.3-39.4	0.31
Body-weight (kg)	65.3	59.5-71.1	70.3	67.5-72.9	67.1	64.0-70.3	0.19
Height (cm)	155.9	152.6-159.2	158.9	157.9-159.9	155.1	153.4-156.9	0.001*
Height (cm)	155.9	152.6-159.2	158.9	157.9-159.9	155.1	153.4-156.9	0.001*
BMI† (kg/m^2^)	26.9	24.5-29.3	27.8	26.7-28.8	28.2	26.5-29.8	0.69
EI‡ (kcal/day)	1,835.4	1,643.7-2,027.2	2,454.5	2,323.3-2,585.7	2,004.6	1,791.7-2,217.5	0.0001*
RMR§ (kcal/day)	1,470.5	1,356.5-1,584.5	1,496.3	1,441.7-1,550.9	1,363.4	1,287.5-1,439.2	0.06
EI:RMR	1.3	1.1-1.4	1.7	1.6-1.8	1.5	1.3-1.7	0.003*
Educational level (%)							
Primary school	4 (14.8)		10 (8)		2 (5.7)		
Guidance school	10 (37)		46 (36.8)		9 (25.7)		0.02
High school	10 (37)		37 (29.6)		11 (31.4)		
University/College	3 (11.1)		32 (25.6)		13 (37.1)		
Reporters (%)							
Underreporter	16 (59.3)		38 (30.4)		12 (35.3)		
Plausible reporter	11(40.7)		76 (60.8)		20 (57.2)		0.05**
Overreporter	0		11 (8.8)		3 (7.5)		

†Body mass index;

‡Energy intake;

§ Resting metabolic rate;

*Significant difference between clusters (one-way ANOVA test);

**Between clusters in all categories (Fisher's exact test)

In this study, we found three clusters, with most people being in the unhealthy cluster, followed by healthy cluster and mixture cluster, and the proportion of underreporters was the lowest among healthy cluster. However, one study in Brazil showed that, among Brazilian women, underreporting of energy intake tends to be more severe among subjects from the healthy cluster (22). Furthermore, Scagliusi *et al*. failed to show significant differences among clusters (22). Nevertheless, underreporters tend to report healthier dietary patterns, and it is also very unlikely that individuals in the cluster with the highest intake of fat and sugar, have EI:RMR <1.35. Wirfalt *et al.* have previously shown that individuals with EI:RMR <1.35 are more likely to be in the healthy cluster, which was characterized by intake of high fibre and low-fat foods (47).

Furthermore, our findings generally agree with a study in Brazil. This study showed that, among Brazilian women, underreporting of energy intake is not uniformly distributed among dietary pattern clusters and tends to be more severe among subjects from the healthy cluster (22).

In the present study, after excluding the underreporters, only 107 subjects were left as plausible reporters and, thus, two dietary patterns were recognized for them (healthy and unhealthy). Our findings are not in agreement with the published results from Geisinger Rural Aging Study (11). Bailey *et al*. (11) identified two dietary patterns for all and plausible reporters, with one pattern being more nutritious compared to the other. They reported that dietary patterns of plausible reporters were not vastly different from all reporters (11). However, although dietary patterns were substantially similar for all and plausible reporters, inconsistencies were noted for seven food-groups. In other words, there was an inconsistency for 28% of the food-groups in their study. We further did cross-tabulation of plausible reporters and assessed the agreement (kappa) regarding healthy and unhealthy pattern for both analyses. The kappa statistic was poor (0.07).

### Limitations

This study has several limitations to consider. The most important is using FFQ for collecting dietary data, which may not truly represent the dietary pattern of individuals and may report some food-groups different from dietary records (fruits, vegetables, and eggs); although the FFQ used was shown to be a valid tool for assessing the dietary pattern of Iranian adults (27). The second limitation is: conducting the study on one gender from one city, which could limit the generalizability to the Iranian population. Another concern is lack of data on physical activity level used in estimating energy expenditure (EE). Therefore, EE was estimated based on a reasonable assumption of minimum physical activity level and the equation RMR×1.35 (12). The main limitation of Goldberg equation is that using EI:RMR ratio for assessing energy intakes requires the knowledge of energy expenditure or requirement. In addition, assessing the physical activity to estimate underreporting rate is another challenge.

### Conclusions

The rationale for this study is that underreporting is more prevalent for some specific food-groups; nevertheless, the present study has identified underreporters according to the RMR equations. A positive point of the present study is using the FitMate which could yield more reliable RMR levels in study participants (32). Strength of this study is the extraction of the dietary patterns and comparing the different dietary patterns in relation to status of energy reporting in a developing country. To our knowledge, this is the first study in a Middle Eastern country to concern the effect of underreporting on food patterns and also the first in Iran to concern the relationship between dietary patterns and energy reporting errors.

This study contributed to the mounting evidence that suggests characteristics of subjects (i.e. age and weight status) to be related to reporting errors. These factors should be used in controlling for or taken into account in the statistical models when examining relationships between diet and health. The unique contribution of this study is that, due to errors in reporting dietary intake, dietary patterns might not be generally valid. Errors in reporting dietary intake is some sort of information bias. This bias may confound conclusions and may lead researchers to draw incorrect conclusions. To avoid these, we might have 2 options. First, we could run the dietary pattern analysis two times—one time for all subjects and one time for just plausible reporters. Then, we can compare the results. Second, adjustment for under- and overreporters in absence of improved data might reduce the bias in association between food-group intakes or dietary patterns and health-related outcomes.

More investigation is necessary to find how reporting errors affect dietary pattern analysis and also what the best way to validate this technique is, especially before dietary recommendations are made based on these.

## ACKNOWLEDGEMENTS

Authors would like to thank the subjects for their enthusiastic support. We also thank the members of the National Nutrition and Food technology Research Institute for their kind collaboration. This work was supported by the National Nutrition and Food Technology Research Institute of Shahid Beheshti University of Medical Sciences and Health Services.
